# Iontophoretic delivery of caspofungin acetate to the cornea and sclera and its intracorneal biodistribution

**DOI:** 10.1016/j.ijpx.2025.100451

**Published:** 2025-11-19

**Authors:** Laura Gisela González Iglesias, Yogeshvar N. Kalia

**Affiliations:** a School of Pharmaceutical Sciences, University of Geneva, 1211 Geneva, Switzerland; b Institute of Pharmaceutical Sciences of Western Switzerland, University of Geneva, 1211 Geneva, Switzerland

**Keywords:** Caspofungin, Ocular delivery, Iontophoresis, Transscleral, Cornea, Keratitis

## Abstract

Caspofungin (CAS) is a potent antifungal agent belonging to the echinocandin family. It is a water-soluble dication at physiological pH, making it a good candidate for iontophoresis. Intracorneal iontophoretic delivery and biodistribution of CAS and its electrically assisted transport into the sclera were investigated as a function of experimental conditions, including donor concentration (1, 5 and 10 mg/mL) and application time (5 and 20 min) using an in-house set-up (with Ag/AgCl electrodes) and a marketed iontophoretic applicator (Iontofor CXL®) that used inert (stainless steel) electrodes. CAS deposition after passive delivery for 20 min (10 mg/mL) was 64.9 ± 23.7 μg/cm^2^ and 370.9 ± 67.49 μg/cm^2^ in the cornea and sclera, respectively. This increased by ∼14- and 3-fold, respectively, after iontophoresis at current densities of 1.5 mA/cm^2^ and 3.5 mA/cm^2^, respectively, for corneal and scleral application using the in-house set-up. The same trends were observed in the Iontofor CXL® studies – although the superiority of iontophoresis over passive delivery was less pronounced due to the electrolysis of water at the anode and the creation of competing hydroxonium ions in the anodal compartment. Intracorneal biodistribution studies showed that after iontophoresis using the Iontofor CXL® (1 mA, 20 min) significantly greater amounts of CAS were present in each lamella as compared to passive delivery and higher CAS concentrations were also achieved in the stroma and endothelium. CAS concentrations in the epithelium, stroma and endothelium after short-duration iontophoresis (1 mA for 5 min) – were > 100-fold higher than the MIC_90_ reported for *Candida albicans* and *Aspergillus* spp.

## Highlights

• Demonstrated the feasibility of using iontophoresis for the noninvasive intracorneal and scleral delivery of caspofungin and its superiority to passive administration.
• Determined the post-iontophoretic spatial distribution of caspofungin in the anatomical regions of the cornea.
• Confirmed that intracorneal concentrations after iontophoresis for 5 min were > 100-fold higher than the MIC_90_ reported for *Candida albicans* and *Aspergillus* spp.

## Graphical Abstract

**Figure:**
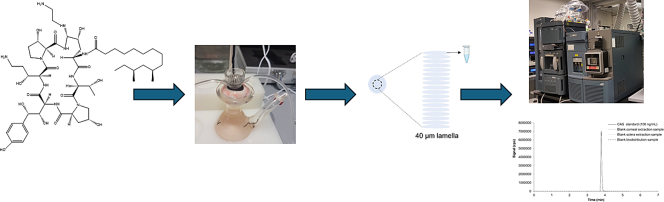

## 1 Introduction

Ocular mycoses give rise to severe ocular morbidity and loss of vision (Rammohan et al., 2020; Steinmetz et al., 2021). *Candida albicans* is the most frequently isolated yeast in the developed world and is often associated with chronic and complicated ocular surface disease (Sun et al., 2007). Filamentous fungi are the principal causes of mycotic keratitis and the most common etiological agents of exogenous fungal endophthalmitis in most parts of the world, with either *Fusarium* spp. or *Aspergillus* spp. being the commonest isolates (Rammohan et al., 2020; Tanure et al., 2000). These infections can be localized in ocular tissues such as the cornea, aqueous humour, sclera, vitreous humour, and conjunctiva (Thakkar et al., 2019) and successful therapy requires penetration of the antifungal drug into the relevant tissues and sub-compartment(s) of the eye, i.e., cornea, aqueous humour (for keratitis), vitreous humour (for endophthalmitis) (Felton et al., 2014).

**Fig. 1:**
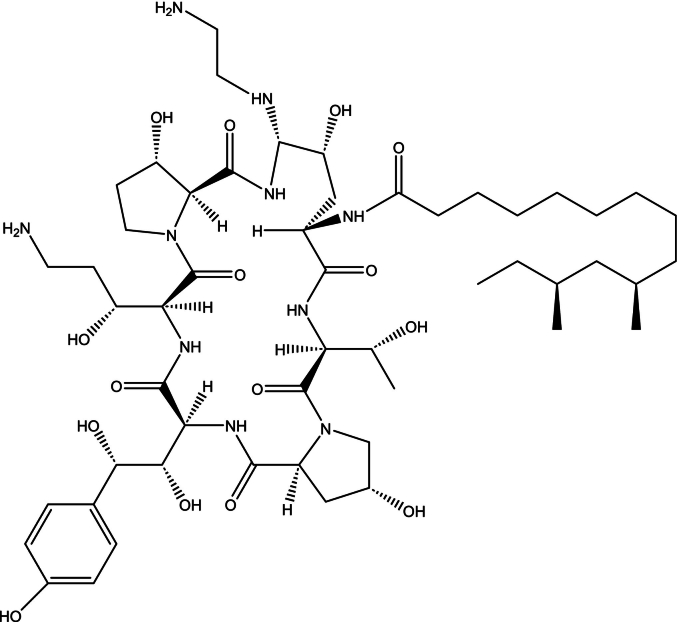
Chemical structure of caspofungin (MW = 1093.1 Da; log D_pH 7.4_ = −7.58 and log D_pH 5.5 =_ − 10.08 (Chemaxon Playground v.1.6.2 (2025)); pKa 9.46, 9.76 (Uribe et al., 2019)).

Caspofungin (CAS) belongs to the newer class of semisynthetic amphiphilic lipopeptides (Fig. 1) that specifically inhibit β-(1,3)-D-glucan synthase leading to cell lysis due to increased cell wall permeability (Hoang, 2001; Maharana et al., 2016; Wang et al., 2015). CAS exhibits potent in vitro and in vivo antifungal activity against *Candida albicans* (MIC_90_ = 0.6 μg/mL) and *Aspergillus* spp. (MIC_90_ = 0.5 μg/mL) (Neoh et al., 2014), including pathogens resistant to azoles or amphotericin B (Hoang, 2001).

Topical natamycin (administered as eye-drops) is the first-line therapy to treat fungal infections in the anterior segment. However, ∼95 % of the topically applied drug is quickly washed out, reducing its concentration and residence time – when combined with limited drug permeation due to the corneal transport barrier, the result is poor ocular bioavailability (Ansari et al., 2013; Neoh et al., 2014; Thakkar et al., 2019). Multiple applications are required to reach and to maintain therapeutically effective concentrations, and in many cases combination with orally or intravenously administered antifungal agents is needed (Ansari et al., 2013; Gratieri et al., 2017; Souto et al., 2019).

In the case of posterior segment infections, the treatment consists primarily in the use of systemically administered antifungals and depending on the intraocular structures affected it can also be necessary to use more invasive treatments such as intracameral or intravitreal injection or surgery (penetrating keratoplasty or vitrectomy) (Maharana et al., 2016; Rammohan et al., 2020; Riddell et al., 2011). However, systemically administered drugs have poor ocular bioavailability requiring the use of higher doses with a consequent increase in the risk of off-target adverse effects (Felton et al., 2014) while the possible risks associated with injection into the eye cavity are retinal detachment, traumatic cataract, and vitreous haemorrhage (Falavarjani and Nguyen, 2013; Sampat and Garg, 2010).

CAS has been reported as an alternative treatment for keratitis (Wang et al., 2015) and endophthalmitis (Shen et al., 2014), due to its efficacy and reduced toxicity. However, CAS has a low oral bioavailability (<0.2 %) and its intravenous administration results in an extensive tissue distribution but its ocular bioavailability still remains minimal due to its high level of protein binding and high molecular weight (Felton et al., 2014; Shen et al., 2014). Topical CAS eyedrops (1–5 %) have been used to treat *Candida* keratitis, recurrent infections and in the treatment of fungal keratitis refractory to voriconazole (Goldblum et al., 2005; Hurtado-Sarrio et al., 2010).

To circumvent the limitations of existing treatments, other drug delivery techniques are being tested in order to improve ocular bioavailability and hence the outcome of local treatments for fungal infections. Iontophoresis is a non-invasive technology involving the application of a mild electric potential to enable electromigration of ionized species across biological barriers and this technique has been used to successfully deliver antifungals – ketoconazole and miconazole (Grossman and Lee, 1989; Yoo et al., 2002). Non-invasive transepithelial riboflavin/ultraviolet A induced corneal collagen cross linking (CXL) assisted by iontophoresis is used for the treatment of keratoconus in order to minimize the potential complications created by corneal epithelial debridement. In clinical studies, the use of a transcorneal iontophoretic applicator (Iontofor CXL®, SOOFT Italia S.p.A) improved riboflavin penetration to the stroma and decreased the procedure time (Bouheraoua et al., 2014; Mazzotta et al., 2020). We have previously used the Iontofor CXL® applicator to improve the intracorneal delivery of amino acid prodrugs of triamcinolone acetonide (Santer et al., 2025).

The presence of ionizable groups (imparting a double positive charge at physiological pH) and high aqueous solubility render CAS a good candidate for controlled ocular delivery by transcorneal and transscleral iontophoresis. The objectives of this study were: (a) to develop and to validate an analytical method to quantify CAS deposition in corneal and scleral tissue, (b) to explore the effect of experimental parameters on the iontophoretic transport of CAS in vitro using our in-house set-up (with Ag/AgCl electrodes) and the marketed Iontofor CXL® applicator (with stainless steel electrodes), (c) to study the post-iontophoretic intracorneal biodistribution profile of CAS using the Iontofor CXL® system, and (d) to evaluate the feasibility of delivering therapeutically relevant amounts of CAS.

## 2 Materials and methods

### 2.1 Materials

Caspofungin diacetate (MW = 1213.42 Da) was purchased from Hangzhou Dayangchem Co., Ltd. (Hangzhou, China), Dulbecco's Phosphate Buffered Saline and MES monohydrate were purchased from Sigma-Aldrich (Buchs, Switzerland). Sodium chloride was obtained from Fisher Scientific (Loughborough, UK). Silver wire and silver chloride (AgCl) for the fabrication of electrodes were purchased from Sigma Aldrich (Steinheim, Germany). PVC tubing (3 mm ID, 5 mm OD, 1 mm wall thickness) and agarose used to prepare salt bridge assemblies were obtained from VWR International AG (Dietikon, Switzerland) and Conda (Madrid, Spain), respectively. Acetonitrile (ACN) and methanol (MeOH) were HPLC grade (Fischer Scientific (Loughborough, UK). Formic acid (FA; 99 % extra pure) was obtained from Biosolve (Dieuze, France).

All aqueous solutions were prepared using ultra-pure water (Purelab ultra, ELGA, resistivity >18 MΩ.cm). All other chemicals were at least of analytical grade. Power supply (I-ON CXL®; SOOFT Italia S.p.A.) and corneal application system (Iontofor CXL®, SOOFT Italia S.p.A.) were kindly provided by SOOFT Italia S.p.A.

### 2.2 Analytical method development

A robust UHPLC-MS/MS method was developed and validated for the quantification of caspofungin acetate deposition in ocular tissues. The liquid chromatographic system consisted of a Waters ACQUITY UPLC® core system (Baden-Dättwil, Switzerland) including a binary solvent manager, a sample manager with an injection loop volume of 10 μL and a column manager. Gradient separation was performed using an ACQUITY UPLC® BEH C18 column, 1.7 μm, 25 × 2.1 mm, attached to an ACQUITY UPLC® BEH C18 Van Guard™ Pre-column, 1.7 μm, 5 × 2.1 mm. The mobile phase consisted of 0.1 % formic acid (*v*/v) (Phase A) and 0.1 % formic acid in ACN: MeOH (80:20 v/v) (Phase B) with a flow rate of 0.15 mL/min and a runtime of 7 min. Column temperature was kept at 40 °C and the sample manager was at 12 °C. Injection volume was set at 5 μL (partial loop injection mode). The mass spectrometry (MS) system consisted of a Waters XEVO® TQ-S Micro tandem quadrupole detector (Baden Dättwil, Switzerland) fitted with a *Z*-spray electrospray ionization source. MS detection was performed using electrospray ionization in the positive-ion mode (ESI+) and multiple reaction monitoring (MRM). The detection settings for CAS are presented in Table 1. Data processing was performed using Waters MassLynx software version 4.1. The peak for CAS was observed at 3.9 min. The LOD (limit of detection) and LOQ (limit of quantification) were 3.3 and 10 ng/mL, respectively. The method was validated according to ICH guidelines (including with respect to specificity (**Fig. S1**) and intra- and inter-day accuracy and precision (**Table S1**)) and complete details are provided in the **Supplementary Information**.

**Table 1:** MS settings for the detection of CAS.

Parameters	Values	
Nature of the parent ion	[M + 2H]^2+^	
Precursor ion (*m*/*z*)	547.39	
Product ion (m/z)	538.50	137.09
Collision energy (V)	8	34
Cone voltage (V)	7	
Capillary voltage (kV)	3.6	
Desolvation temperature (°C)	350	
Desolvation gas flow (L/h)	650	
Cone gas flow (L/h)	0	
LM resolution 1	9.7	
HM resolution 1	15.1	
Ion energy 1 (V)	−0.6	
LM resolution 2	9.8	
HM resolution 2	15.0	
Ion energy 2 (V)	0.6	

### 2.3 Preparation of ocular tissues

The porcine eye has been histologically characterized and is generally accepted as a representative anatomical model for the human eye (Nicoli et al., 2009; Pescina et al., 2015). Fresh porcine eyes were obtained immediately after the sacrifice of the animals (40–60 kg) from a local abattoir (Abattoir de Loëx Sarl; Loëx, Switzerland). Eye globes were first rinsed with saline solution to remove any remaining blood, followed by the removal of adherent muscle with a scalpel prior to the transport studies. The intact eye globes were used for the transport studies using the Iontofor CXL® system. For the experiments using excised tissues, the cornea was separated by excision along the scleral limbus, and the iris-ciliary body and crystalline were separated gently from the cornea using forceps, this tissue was used for transport studies within 3 h of harvesting. The sclera was obtained by removing the iris-ciliary body, lens, and vitreous humour. The eye globe was cut into two halves, the choroid/retina tissue underlying the sclera was removed using a cotton swab, the anterior sclera was used for permeation experiments.

### 2.4 Stability studies

#### 2.4.1 Stability of CAS in the presence of cornea and sclera

The effect of porcine cornea and sclera on CAS stability in PBS pH 7.4 solution was assessed. Cornea and sclera were cut into small pieces and were collected separately in a Falcon tube and 10 mL of PBS solution (pH 7.4) was added. The tubes were vortexed for 1 h at room temperature, followed by centrifugation at 5000 rpm for 10 min. The supernatant was used to investigate the stability of CAS in contact with ocular tissue matrix. An aliquot of a stock of CAS (1.5 mg/mL, 0.2 mL) was added into the supernatant (9 mL). The solution was stirred and kept at 32 °C for 90 min, aliquots were withdrawn at pre-determined time points. All experiments were performed in triplicate.

#### 2.4.2 Stability in the presence of an electric current

The impact of electric current on the stability of a CAS solution (1 mg/mL) was evaluated prior to the iontophoretic permeation studies: (a) 1 mL of unbuffered CAS solution (1 mg/mL, pH 8.05), (b) 1 mL of CAS in PBS, and (c) 1 mL of CAS in 10 mM MES buffer pH 5.5, were evaluated using salt bridges in the presence of the highest current density used in the transport experiments (i.e., 3.5 mA/cm^2^). Samples were collected and analyzed every 5 min for a period of 30 min. Experiments were performed in triplicate.

### 2.5 Iontophoretic transport studies

#### 2.5.1 Experimental protocol using “lab” set-up

For the delivery experiments, the isolated cornea/sclera was clamped into vertical Franz diffusion cells (Glass technology; Geneva, Switzerland), with the corneal epithelium and episcleral layer facing the donor compartment (area = 0.8 cm^2^). For iontophoretic experiments, conventional silver/silver chloride (Ag/AgCl) electrodes were added to the set-up described above. The anode (Ag) was connected to the donor compartment containing 1 mL of CAS solution (solution in 10 mM MES; pH 5.5) via saline bridges (3 % agarose+0.1 M NaCl) to reduce the competition from sodium ions. The cathode (AgCl) was directly inserted in the receiver compartment (PBS pH 7.4; 2 mL) through the sampling arm. The current density was applied using a constant current power generator (APH 1000 M, Kepco Inc.; Flushing NY, USA). The passive permeation experiments were performed using the same set-up but without current application and served as controls. At the end of the transport experiments, a 1 mL aliquot was withdrawn from the receiver compartment. The system was immediately disassembled, and the surface of the tissue was washed under running water for 30 s. CAS was extracted from the sclera and cornea post-iontophoresis by cutting the tissue into small pieces and soaking them in 1 mL 1 % FA in MeOH/water mixture (2:1) for 4 h. The extraction procedure was validated, and the CAS recovery efficiency was >90 % (**Table S2**; complete details in the **Supplementary Information**). The supernatant was filtered using 0.20 μm polyvinylidene difluoride (PVDF) syringe filters (Macherey-Nagel; Düren, Germany), the samples were analyzed using UHPLC-MS/MS. Experiments were performed with at least four replicates.

The effect of CAS concentration (1, 5 and 10 mg/mL in 10 mM MES, pH 5.5) on iontophoretic transport was investigated. The current was applied for 20 min and current densities of 1.5 mA/cm^2^ and 3.5 mA/cm^2^ were used for the corneal and scleral iontophoretic delivery experiments, respectively.

#### 2.5.2 Experimental protocol using the SOOFT system set-up

CAS delivery studies were performed on whole eye globes using a commercially available corneal applicator system (area = 0.636 cm^2^) and power supply (Iontofor CXL®, SOOFT Italia S.p.A). The polarities of the system were made convertible by inserting wire connectors, as previously reported (Santer et al., 2025). Porcine eyeballs were positioned with the cornea side up in custom-made vertical Franz diffusion cells. (Glass Technology; Geneva, Switzerland) and the receiver compartment was filled with PBS solution. The SOOFT eye cup, which contains the positive electrode, was fixed with an annular suction ring on the corneal surface and the return electrode (obtained by dismantling the patch applicator) was inserted in the receiver compartment.

The first set of experiments investigated the effect of concentration (1, 5 and 10 mg/mL; in 10 mM MES, pH 5.5) on CAS corneal delivery. The current and application time were fixed at 1 mA and 20 min (5 min × 4 times), respectively. The second set of experiments studied the effect of application time – 5 and 20 min – on CAS corneal delivery using a current intensity of 1 mA and a 5 mg/mL CAS solution.

The intracorneal CAS biodistribution profiles were also determined after both passive and iontophoretic delivery. After completion of the experiments, the formulation application area was cleaned, and the central corneal section (area = 0.283 cm^2^) was isolated and fixed on a cork base using O.C.T. mounting media. The tissue was snap-frozen in isopentane cooled with liquid nitrogen. The tissue sample was mounted on a cryotome (Thermo Scientific CryoStar NX70; Walldorf, Germany) sample holder. Seventeen 40 μm thick lamellae were prepared; each lamella and the remaining corneal tissue (with a thickness of up to ∼200 μm) were placed separately in individual Eppendorf tubes. CAS deposited in each lamella was extracted overnight using 200 μL of 1 % FA MeOH/Water (2:1) mixture and analyzed by UHPLC-MS/MS.

### 2.6 Statistical analysis

A minimum of 4 replicates were performed for each experiment. Data are expressed in terms of the mean ± standard deviation. Data and statistical analyses were performed using Graph Pad Prism 9.0 Software (San Diego, USA). A Grubbs' test with an α of 0.05 was used to identify outliers. The results were evaluated statistically using nonparametric analysis, Mann-Whitney test was used to compare two data sets. The level of significance was fixed at *p* < 0.05.

## 3 Results and discussion

### 3.1 Stability studies

CAS was stable in the presence of porcine cornea and sclera with a recovery of 97.9 % ± 1.9 % and 99.9 % ± 4.9 %, respectively. After application of current for 20 min, the concentration of CAS was found to be 82 % ± 1.8 % in water, 87 % ± 0.5 % in MES, and 91 % ± 1.3 % in PBS, of the initial values. These results suggested that CAS solution might have been slightly affected by the current in the absence of salt in the solution (water and MES solution as compared to the PBS solution).

### 3.2 Iontophoretic delivery of CAS to the cornea using the “lab” set-up with Ag/AgCl electrodes

**Fig. 2:**
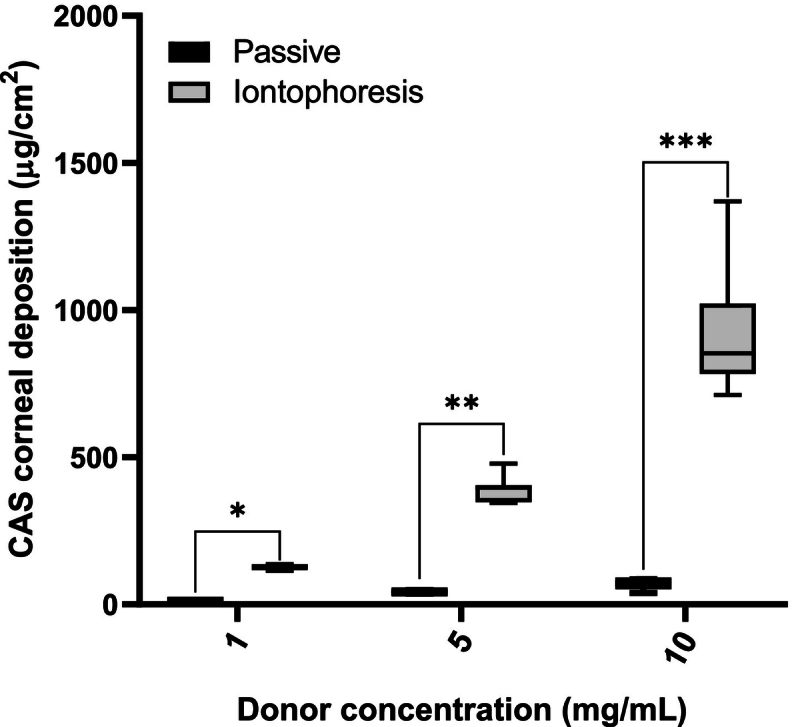
Comparison of CAS corneal deposition from solution (1, 5 and 10 mg/mL) after passive and iontophoretic (at 1.5 mA/cm^2^) delivery for 20 min. (Mean ± SD; *n* > 3). *p = 0.0159, **p = 0.0061, ***p = 0.0121.

To investigate the benefit of current application on the corneal delivery of CAS, its deposition into and permeation across the tissue were determined after current application for 20 min. Corneal deposition of CAS as a function of donor concentration is illustrated in Fig. 2. The corneal deposition for the passive “control” experiments was 8.0 ± 2.6 μg/cm^2^, 42.0 ± 5.6 μg/cm^2^ and 64.9 ± 23.7 μg/cm^2^ from 1, 5, and 10 mg/mL CAS solutions, respectively.

CAS displayed poor passive penetration into intact cornea, consistent with it being a large (MW 1093.1 Da) and highly hydrophilic molecule. The corneal epithelium represents the principal barrier to transcorneal transport, and permeability is limited by hydrophilicity and molecular weight – compounds with MW >500 Da have difficulty penetrating the intact corneal epithelium. The stroma and endothelium, on the other hand, contribute very little to the tissue resistance to transcorneal permeation; due to their high water content, they only inhibit permeation of highly lipophilic compounds (Mun et al., 2014). The poor passive permeation of CAS observed here was also consistent with a previous report of patients receiving continuous topical administration with 0.5 % CAS (1 drop every hour for four hours) where low drug concentrations (30–90 ng/mL) were achieved in the aqueous humour (Neoh et al., 2011). The effect of an impaired corneal epithelium on the ocular penetration of CAS was observed in a rabbit model using a 0.7 % CAS solution, 1 drop was administered every 30 min for 6 h, CAS was not detected in the aqueous humour of rabbits with an intact corneal barrier while the abrasion of corneal epithelium enabled quantifiable levels of CAS to reach the aqueous humour (4.94 ± 1.80 μg/mL) (Vorwerk et al., 2009). Anodal corneal iontophoresis at 1.5 mA/cm^2^ of buffered 1, 5 and 10 mg/mL CAS solutions resulted in statistically significant increases in corneal deposition – 127.0 ± 9.5 μg/cm^2^, 393.0 ± 45.2 μg/cm^2^ and 926.8 ± 251.2 μg/cm^2^ – corresponding to 16-, 9- and 14-fold increases with respect to the corresponding passive controls. Regression analyses confirmed that the deposition of CAS increased linearly with concentration (R^2^ = 0.98).

Previous studies show that the transdermal iontophoretic flux of drugs (i.e. lidocaine, propranolol, rasagiline, glycyrrhizin) is independent of the donor concentration in the single-cation situation, while the efficiency of drug delivery is strongly dependent on concentration in the presence of electrolytes and in some cases also in the presence of an accompanying buffer. The absence of background electrolytes is the desired condition as it allows us to achieve the highest flux without increasing the drug concentrations and the obtained flux will be dependent only on the relative mobility of the drug and counterion. However, pharmaceutical formulations often require the incorporation of additives (i.e. preservatives, buffering agents) which introduce external co-ions (Kalaria et al., 2012; Marro et al., 2001; Yamamoto et al., 2013). In the present set-up, the use of salt bridges avoided the presence of Na + ions in the donor compartment, and CAS solutions were prepared using MES buffer (zwitterionic); however, a possible competition from this buffer under asymmetric donor-receiver ion concentrations has been reported previously (Kalaria et al., 2013), which can explain the linear relation between CAS corneal deposition and the CAS concentration in the donor compartment.

Although CAS concentrations in the receiver compartment were below the LOD after 20 min iontophoresis at all of the conditions tested, the results were extremely promising, since the cornea is the site of infection in fungal keratitis and is the primary target tissue (Rammohan et al., 2020). Furthermore, the accumulation of CAS in the cornea can be an option to create an in situ drug depot to enable a subsequent sustained drug release into the aqueous humour.

### 3.3 Iontophoretic delivery of CAS to the sclera

**Fig. 3:**
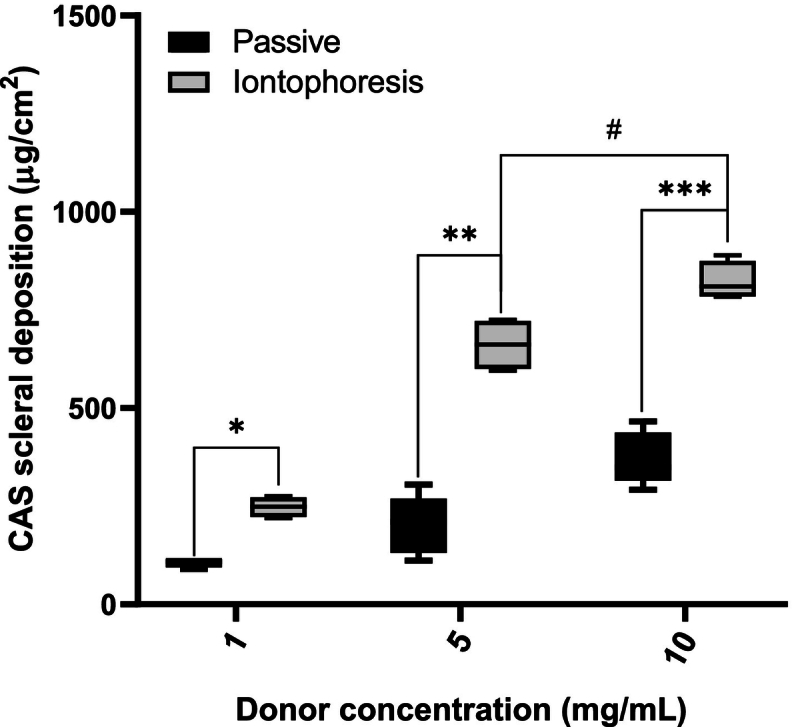
Comparison of CAS scleral deposition from solution (1, 5 and 10 mg/mL) after passive and iontophoretic (at 3.5 mA/cm^2^) delivery for 20 min. Mean ± SD; *n* > 4). *p = 0.0286, **p = 0.0028, ***p = 0.0159, ^#^*p* = 0.0286.

The sclera represents the outermost and barrier to drug transport to the back of the eye. The maximum scleral deposition of CAS after passive application for 20 min was 370.9 ± 67.49 μg/cm^2^ (observed with the 10 mg/mL CAS solution). For comparison, iontophoresis produced a significantly higher CAS deposition – 248.0 ± 26.1 μg/cm^2^, 661.0 ± 65.9 μg/cm^2^, 822.7 ± 49.1 μg/cm^2^ for 1, 5 and 10 mg/mL, respectively (Fig. 3). Comparison of the passive permeation of CAS through cornea and sclera clearly illustrates how the nature of the ocular tissue affects transport. Nevertheless, after transscleral iontophoresis for 20 min, CAS was not detected in the receiver compartment.

The increase in CAS delivery using transscleral iontophoresis over passive transport was not as significant as that obtained for corneal delivery. Three main transport mechanisms have been reported to be responsible for drug transport when iontophoresis is applied: passive diffusion, electroosmosis (EO) and electromigration (EM) (Gratieri et al., 2017; Kalia et al., 2004). For transdermal delivery, the passive flux of hydrosoluble, ionized drugs through the skin is negligible due to the presence of the stratum corneum, a very effective permeability barrier; however, the corneal epithelium and in particular, the episcleral layer are less restrictive (Gratieri et al., 2017). During transscleral diffusion, molecules permeate through porous spaces within a collagen aqueous network, so hydrophilic molecules such as CAS are more likely to go through the sclera (Nicoli et al., 2009; Prausnitz and Noonan, 1998). Fig. 3 shows that for CAS transscleral delivery the passive component makes an appreciable contribution to total delivery.

A statistically significant effect with respect to tissue deposition was observed when the CAS donor concentration was increased from 1 to 5 mg/mL; however, when a 10 mg/mL solution was used, although the deposition was statistically higher (p = 0.0286), it was not proportional to the concentration increase (Fig. 3**)**. This behaviour has been observed also for transscleral delivery of the cationic peptide vancomycin and has been attributed to association with negatively charged proteoglycans present in the scleral matrix, this interaction also leads to accumulation of the positively charged drug in the membrane, reducing the permselectivity thereby resulting in depression of electroosmotic flow (Gungor et al., 2010).

### 3.4 Iontophoretic delivery of CAS to the cornea and ocular biodistribution using the Iontofor CXL device

**Fig. 4:**
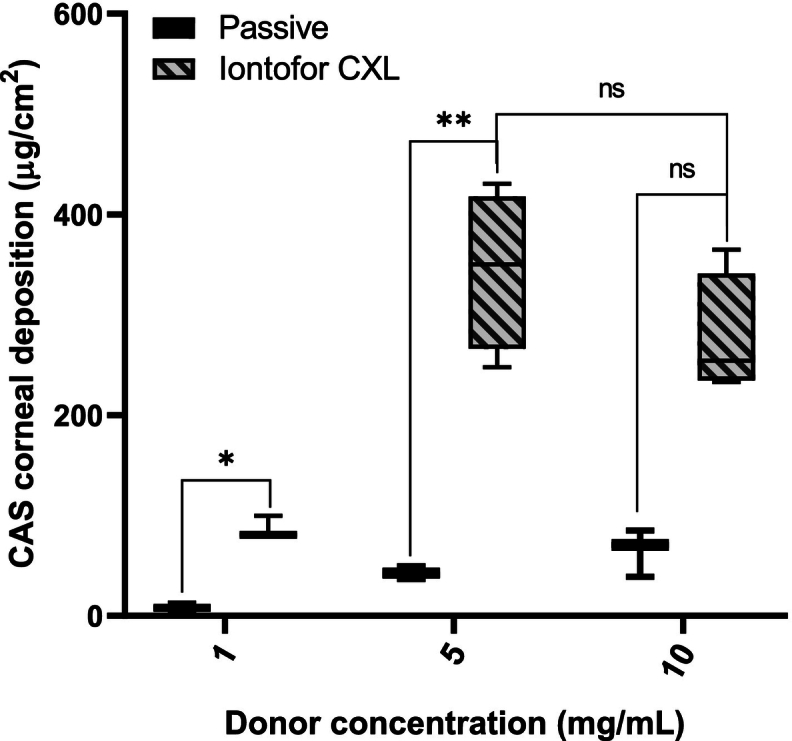
Comparison of CAS corneal deposition from solution (1, 5 and 10 mg/mL) after passive and iontophoretic delivery (using the Iontofor CXL device) for 20 min. Mean ± SD; *n* > 3), ns = no statistically significant difference (*p* > 0.05), *p = 0.0357, ** *p* = 0.0286.

Due to the promising results obtained using excised cornea and the lab “set-up” it was decided to investigate CAS delivery using the marketed Iontofor CXL corneal applicator with the same conditions tested above (i.e. concentration and application time) as this device is approved for use in the clinic. Fig. 4 presents the results obtained for corneal deposition of CAS after 20 min treatment using a current density of 1.57 mA/cm^2^. The amounts deposited corresponded to 11-, 8- and 4-fold higher tissue deposition than those seen when using the passive conditions for CAS concentrations of 1, 5 and 10 mg/mL, respectively.

The corneal deposition obtained with the Iontofor CXL system was similar to those obtained previously when using the “in-house” iontophoretic set up for 1 mg/mL (86.8 ± 11.3 μg/cm^2^) and 5 mg/mL (344.8 ± 78.6 μg/cm^2^) CAS in the donor solution; however, there was no increase in CAS deposition upon use of the 10 mg/mL solution (276.8 ± 61 μg/cm^2^). The main differences between the lab set-up and the Iontofor CXL device are (a) the type of electrodes used (Ag/AgCl and inert stainless-steel electrodes, respectively) and (b) the use of a salt bridge to connect the electrode to the formulation as opposed to the electrode being directly in contact with the formulation. During operation of the iontophoretic device (Iontofor CXL), electrolysis of water occurs at the stainless steel electrodes, and highly mobile species are formed: hydronium (H_3_O^+^) ions at the anode and hydroxide ions (OH^−^) are produced at the cathode, since their concentrations will increase with application time, they can compete with the drug (Foschini et al., 2012). The initial pH of the donor compartment solution was 5.5 ± 0.06 and after 20 min iontophoresis application was 4.91 ± 0.12. The use of MES buffer can reduce pH changes caused by the ions generated – decreasing potential pH related effects on stability – but this still introduces other competing ions into the system.

**Fig. 5:**
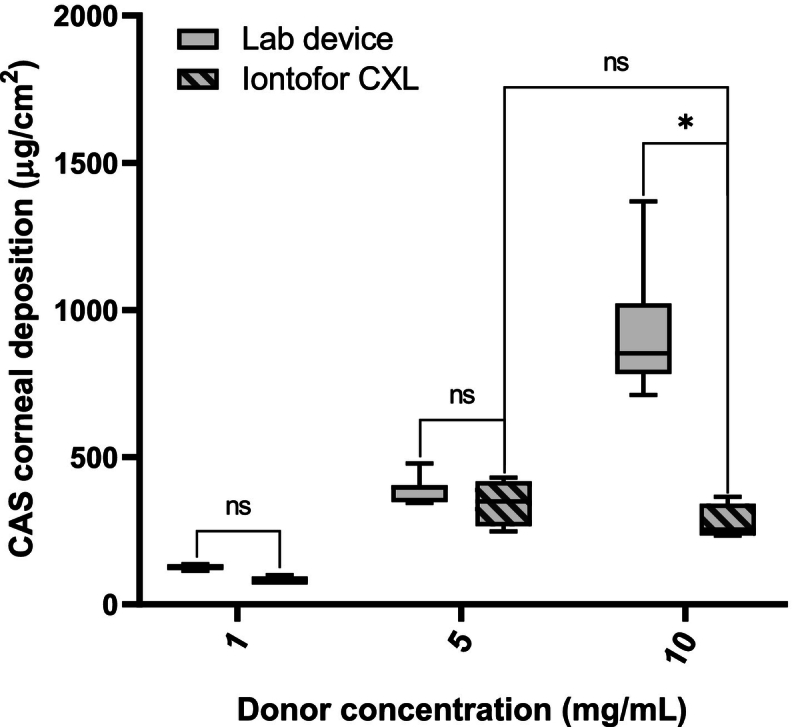
Comparison of CAS corneal deposition from solution (1, 5 and 10 mg/mL) using an in-house experimental set up and the Iontofor CXL device for 20 min (Mean ± SD; n > 3). *P*-values calculated using Mann-Whitney rank test; ns = no statistically significant difference (p > 0.05), * *p* = 0.0040.

Fig. 5 show the corneal deposition obtained with the lab set-up in comparison to the results obtained with the Iontofor CXL applicator, under the conditions tested (current applied = 1 mA, time = 20 min) the concentration of the co-ions (H_3_O^+^) produced in the donor compartment was calculated to be 12 mM which is higher than the CAS concentrations used (5 mg/mL = 4 mM, 10 mg/mL = 8 mM). As the concentration of H_3_O^+^ increases, it will carry an increasing fraction of the total current. Thus, the fraction of current carried by CAS ions will be reduced; as observed previously, increasing the concentration of CAS under these conditions will increase delivery until a plateau is reached. The results highlight the impact of the choice of electrode material and electrochemistry on iontophoretic delivery efficiency. It is also important to consider the effect of cationic CAS species binding to the negative charges present in the transport pathway in the cornea, which could also impact on the contribution of electroosmotic transport to CAS electrotransport.

**Fig. 6:**
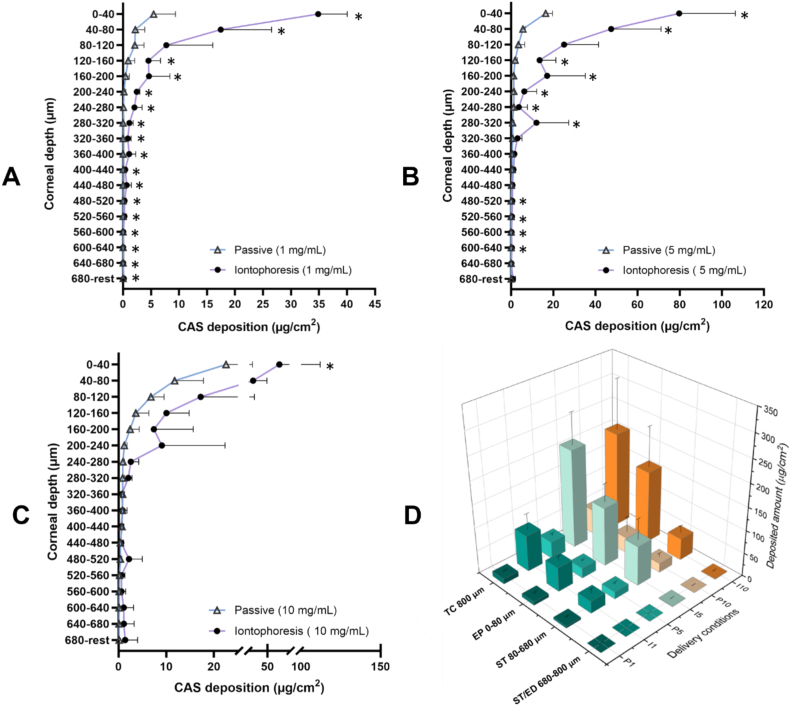
Biodistribution profile of CAS in porcine corneal lamellae (17 × 40 μm + 1 × 120 μm) after passive () and iontophoretic (•) application for 20 min using (**A**) 1, (**B**) 5 and (**C**) 10 mg/mL CAS solution (mean + SD, *n* > 3). (**D**) CAS biodistribution as a function of position in the anatomical corneal regions (TC: total cornea, EP: Epithelium, ST: Stroma and ST/ED: lower stroma/endothelium) after passive (P1, P5 and P10) and iontophoretic (I1, I5 and I10) application of CAS (1, 5 and 10 mg/mL) for 20 min (mean + SD, n > 3). *P*-values calculated using Mann-Whitney rank test; statistically significant differences are denoted by asterisks (* *p* < 0.05).

Research into the ocular administration of CAS has been scarce and to our knowledge there are no previous reports on its ocular biodistribution after topical administration. Since a key requirement for successful topical antifungal therapy is the penetration of drug to the site of infection, in the cornea and aqueous humour (Felton et al., 2014), it was decided to determine and to compare the corneal biodistribution profiles of CAS (18 lamellae: 17 × 40 μm + 1 × 220 μm) after passive and iontophoretic delivery (20 min) (Fig. 6). These corneal biodistribution profiles confirmed the ability of iontophoresis to deliver more CAS into the deeper layers as compared to passive diffusion. In fact, after application for 20 min, CAS deposition was significantly higher in almost every corneal lamella (Fig. 6A-C).

Keratitis can involve the epithelium (e.g., dendritic keratitis), the stroma or interstitium (e.g., interstitial keratitis and deep-seated mycoses), or both as an infiltrate or ulceration (e.g. *Pseudomonas* keratitis). This makes it very important to quantify the antifungal concentrations in the different corneal layers. CAS deposition was also represented as a function of position in the anatomical regions of the cornea (corneal epithelium, stroma, and upper stroma + endothelium) and in total cornea – the superiority of the use of iontophoresis compared to the passive delivery was even more obvious (Fig. 6D).

CAS deposition is mainly found in the epithelium and upper stromal layers, but in the case of iontophoresis, significant amounts were delivered throughout the entire stroma, and small amounts were found in deeper corneal layers. Although the stroma is 80 % water, CAS a highly water-soluble molecule, accumulated in the tissue and did not diffuse freely to deeper layers; it has previously been hypothesized that the presence of interactions with the proteoglycans of the corneal stroma can slow down the diffusion of positively charged CAS to the aqueous humour (Goldblum et al., 2007).

**Fig. 7:**
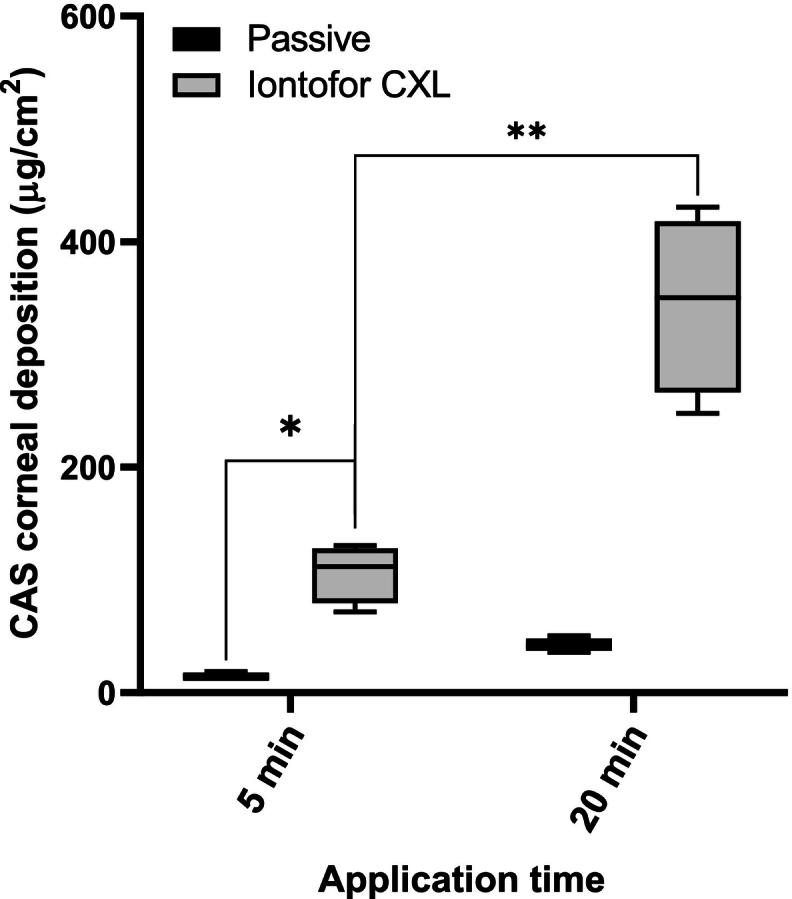
Comparison of CAS corneal deposition from 5 mg/mL CAS solution using passive and iontophoretic delivery (Iontofor CXL device) for 5- and 20-min applications (*n* = 4). *P*-values calculated using Mann-Whitney rank test; statistically significant differences are denoted by asterisks * *p* = 0.0159 and **p = 0.0286.

The intended use of the Iontofor CXL applicator system involves a 5 min treatment, which is also a more patient-friendly (clinically appropriate) application time. The effect of application time on drug deposition was investigated using a 5 mg/mL CAS solution (Fig. 7)**.** With the application of iontophoresis under this more patient-friendly condition, the total deposition was 106.5 ± 37.5 μg/cm^2^, which corresponded to a 7-fold increase in the deposition in comparison to the control experiment (14.9 ± 2.5 μg/cm^2^). The drug amount deposited after treatment for 5 min was proportional to the decreased application time, which means that there is a relation between deposition and the duration of iontophoresis when using a 5 mg/mL concentration, this delivery behaviour is very important in the clinic because it can be used to personalize the treatment.

**Fig. 8:**
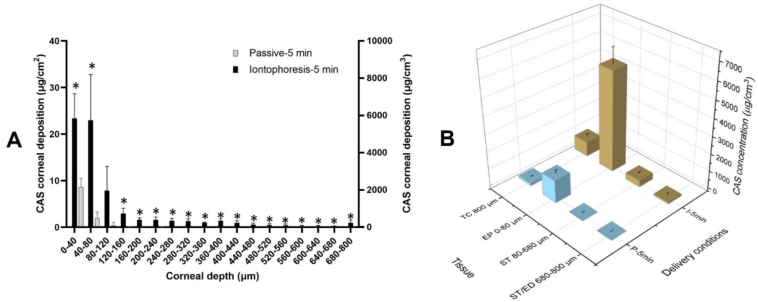
(**A**) Biodistribution profile of CAS in porcine cornea lamellae (17 × 40 μm + 1 × 120 μm) after passive (P5) and iontophoretic (I5) application of a 5 mg/mL solution for 5 min, (**B**) CAS concentrations in cornea (TC) and in the different corneal layers (EP: Epithelium, ST: Stroma and ST/ED: lower stroma/endothelium) after application of iontophoresis (5 mg/mL) for 5 min (mean + SD, *n* > 3). P-values calculated using Mann-Whitney rank test; statistically significant differences are denoted by asterisks (* *p* < 0.05).

The biodistribution profile (Fig. 8A) after this short application demonstrated that iontophoresis for only 5 min considerably enhanced the penetration of CAS to deeper corneal layers in comparison to passive delivery where the drug was mainly deposited in the first few layers.

The use of iontophoresis for a short period enabled deposition of CAS not only in the epithelium (Fig. 8B) but also allowed it to already reach concentrations that exceeded the MIC_90_ reported for the treatment of keratitis caused by *Candida albicans* and *Aspergillus* spp. by >600- and > 150-fold in the stroma and endothelium, respectively.

## 4 Conclusion

For the first time CAS iontophoretic transport was documented and its stability in contact with ocular tissues and under application of electric current was confirmed. The results clearly demonstrated that CAS was an excellent candidate for iontophoresis. The intracorneal deposition of CAS following iontophoresis (1.5 mA/cm^2^) for 20 min displayed a linear dependence over a range of donor concentration from 1 to 10 mg/mL, reaching a maximum deposition of 926.8 ± 251.0 μg/cm^2^ (14-fold higher than passive delivery). In the case of delivery to the sclera, the use of iontophoresis resulted in a 3-fold increase in CAS deposition, in comparison to passive delivery. The use of the Iontofor CXL device in combination with a short application time (5 min) demonstrated potential clinical value by increasing drug deposition by 7-fold as compared to passive delivery in the corneal layers – as evidenced by the biodistribution profiles. Topical CAS treatment could be a targeted alternative to obtain a more effective treatment of keratitis reaching higher drug concentrations in corneal tissues and possibly fewer side effects. Under all the evaluated transcorneal and transscleral conditions, CAS permeation was <LOD. Nevertheless, the deposition of CAS in the cornea and sclera can be considered as a reservoir from which the drug can diffuse to other tissues. As a next step, it would be interesting to evaluate the interaction of CAS with the scleral tissue, and also to investigate the post-iontophoretic biodistribution profile of CAS using the complete eye globe model, where it will be possible to observe the barrier effect of other physical membranes like the choroid, Bruch's membrane, and retina on CAS transscleral drug delivery, before moving to preclinical proof-of-principle studies. The shorter duration of formulation application needed for iontophoresis is a clear advantage over encapsulation-based strategies that require prolonged residence times (and multiple applications) given that uptake depends on passive transport. However, development of a prototype formulation for ocular iontophoresis will require a CAS reservoir system that ensures drug stability. This might involve the development of dry film formulations that are hydrated in situ upon contact with a hydrogel containing the anode prior to iontophoresis (Chen et al., 2016). Therefore, an investigation of the possibilities of using fast dissolving films, xerogels, or other possible “dry formulation” would be an integral part of the next step in the project.

## CRediT authorship contribution statement

**Laura Gisela González Iglesias:** Writing – original draft, Validation, Methodology, Investigation, Formal analysis, Data curation. **Yogeshvar N. Kalia:** Writing – review & editing, Supervision, Resources, Funding acquisition, Formal analysis, Conceptualization.

## Declaration of competing interest

The authors declare that they have no known competing financial interests or personal relationships that could have appeared to influence the work reported in this paper.

## Data Availability

Data will be made available on request.

## Appendix A Supplementary data

Supplementary material Image 1
