# Supplementary material for: Iontophoretic delivery of caspofungin acetate to the cornea and sclera and its intracorneal biodistribution
Source: Int J Pharm X. 2025 Nov 19;10:100451. doi: 10.1016/j.ijpx.2025.100451 (PMC12681843; doi:10.1016/j.ijpx.2025.100451)
Supplement: Supplementary file 1 — Supplementary material [file mmc1.docx]

**SUPPLEMENTARY INFORMATION**

**Iontophoretic delivery of caspofungin acetate to the cornea and sclera and its intracorneal biodistribution**

**Laura Gisela González Iglesias^1,2^, Yogeshvar N. Kalia^1,2*^**

^1^School of Pharmaceutical Sciences, University of Geneva, 1211 Geneva, Switzerland.

^2^Institute of Pharmaceutical Sciences of Western Switzerland, University of Geneva, 1211 Geneva, Switzerland.

*Corresponding author:

School of Pharmaceutical Sciences, University of Geneva,

CMU, 1 rue Michel Servet, 1211 Geneva 4, Switzerland.

Tel.: +41 22 379 3355

Fax: +41 22 379 3360

Email: [yogi.kalia@unige.ch](mailto:yogi.kalia@unige.ch)

1. **Development and validation of UHPLC-MS/MS method for the quantification of caspofungin acetate (CAS).**

The method was validated according to ICH guidelines with respect to specificity, linearity, sensitivity (lower limit of detection LLOD and quantification LLOQ), accuracy and precision.

- 1. Specificity

**Figure S1** presents the chromatograms obtained for CAS standard (100 ng/mL), blank cornea and sclera extraction and blank biodistribution sample. The tissue matrix solutions were used to assess the specificity of the method and evaluate the matrix effect. The method was specific for the quantification of CAS using MRM transition monitored for 547.39→538.50 and 547.39→137.09, CAS eluted at 3.9 min and the peak was clearly separated from the solvent front and corneal and scleral extraction matrix.


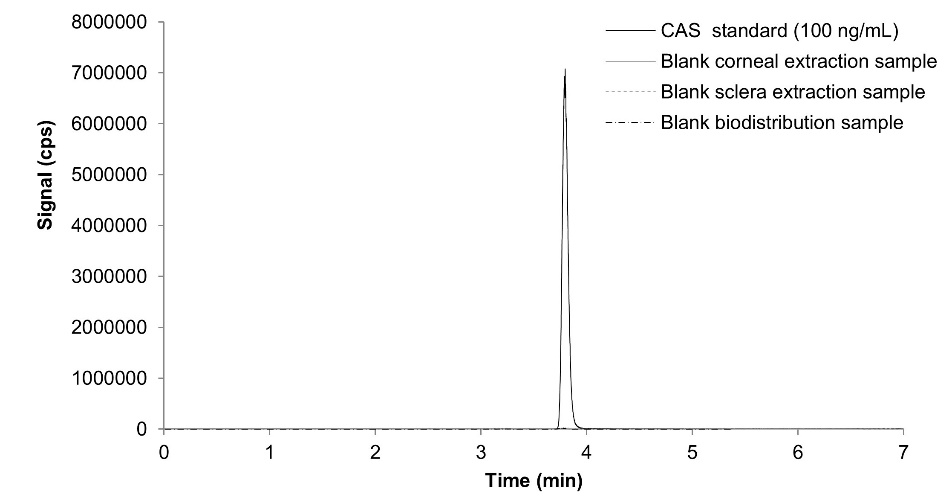


**Figure S1**. Respective MRM traces of CAS standard (547.39→538.50 and 137.09): a) CAS standard in matrix, b) blank corneal extraction sample, c) blank scleral extraction sample and d) blank biodistribution sample.

- 1. Linearity

Calibration curves were constructed by plotting CAS peak area (cps/min) against drug nominal concentration (ng/mL). A good linear fit was found in the concentration range of 10 – 500 ng/mL. Correlation coefficients for all calibration curves were superior to 0.99.

- 1. Limit of detection and limit of quantification

The limit of detection (LLOD) and limit of quantification (LLOQ) was determined using the linear regression equation and found to be 3.3 and 10 ng/mL.

- 1. Accuracy and precision

Accuracy and precision were determined by measuring the intra-day and inter-day recovery and variability of CAS at three different concentrations (10, 100 and 500 ng/mL) over three days. **Table S1** presents the results obtained for intra- and inter-day precision and accuracy. The results indicated that for intra-day measurements, the mean recoveries ranged from 96.4 to 100.1 % (RSD 0.8-2.2 %). The mean recoveries for inter-day analysis on day 1 were between 98.3 and 100.1 (RSD 0.04 and 3.2%) and on day 2, 99.6 and 101 (RSD 1.1 – 2.9%). Considering the ICH (2005) guidelines, the method was considered accurate and precise.

**Table S1**. Intra- and inter-day precision and accuracy for quantification of CAS

| [CAS]_theo_ (ng/mL) | Intra-day | | | Inter-day 1 | | | Inter-day 2 | | |
| --- | --- | --- | --- | --- | --- | --- | --- | --- | --- |
|  | **[CAS]_meas_**  **(ng/mL)** | **RSD (%)** | **Recovery (%)** | **[CAS]_meas_**  **(ng/mL)** | **RSD (%)** | **Recovery (%)** | **[CAS]_meas_**  **(ng/mL)** | **RSD (%)** | **Recovery (%)** |
| 10 | 9.64 ± 0.21 | 2.2 | 100.1 | 9.83 ± 0.31 | 3.2 | 98.3 | 10.10 ± 0.30 | 2.9 | 101 |
| 100 | 99.43 ± 1.06 | 1.1 | 99.4 | 99.71 ± 1.58 | 1.6 | 99.7 | 100.47 ± 2.11 | 2.1 | 100.5 |
| 500 | 500.26 ± 3.89 | 0.8 | 96.4 | 500.37±0.22 | 0.04 | 100.1 | 498.01 ± 5.41 | 1.1 | 99.6 |

1. **Validation of corneal extraction procedure**

The efficiency of the extraction method to recover the antifungal deposited in the corneal tissue was evaluated and validated using porcine cornea samples. Each cornea (n=3) was spiked with a known amount of CAS (in MeOH), the solvent was evaporated completely under the fume hood. Corneal samples were cut into small pieces and soak in the extraction solvent: MeOH/Water (2:1) 1% FA under agitation for 4 h. To determine the best extraction conditions, 2 extraction volumes (0.5 and 1 mL) were tested. After the extraction period, the samples were centrifuge at 10,000 rpm x 10 min and the supernatant was diluted and analysed by UHPLC-MS/MS. The results of the extraction procedure are presented in **Table S2**. The use of 1 mL of extraction volume and agitation for 4 h was selected.

**Table S2.** Validation of CAS extraction from cornea samples using 0.5 and 1 mL of extraction volume.

| Applied amount _theo_  (µg) | Extraction using 0.5 mL | | | Extraction using 1 mL | | |
| --- | --- | --- | --- | --- | --- | --- |
|  | **Applied amount (µg) _meas_** | **Recovered amount**  **(µg)** | **Recovery**  **Efficiency (%)** | **Applied amount (µg) _meas_** | **Recovered amount**  **(µg)** | **Recovery**  **Efficiency (%)** |
| 2 | 2.2 ± 0.03 | 2.0±0.1 | 89.5 ± 4.6 | 2.2 ± 0.03 | 2 ± 0.1 | 88.4 ± 3.7 |
| 7.5 | 7.5 ± 0.2 | 7.1±0.5 | 94.8 ± 6.6 | 7.5 ± 0.2 | 6.9 ± 0.3 | 92.1 ± 4.5 |
| 15 | 15.2 ± 0.9 | 13.1±0.7 | 86.3 ± 4.8 | 15.2 ± 0.9 | 13.7 ± 0.2 | 90.4± 1.3 |
